# Colorectal cancer prevention: Perspectives of key players from social networks in a low-income rural US region

**DOI:** 10.3402/qhw.v11.30396

**Published:** 2016-02-22

**Authors:** Nancy E. Schoenberg, Kathryn Eddens, Adam Jonas, Claire Snell-Rood, Christina R. Studts, Benjamin Broder-Oldach, Mira L. Katz

**Affiliations:** 1 Department of Behavioral Science, University of Kentucky, Lexington, KY, USA; 2 Department of Health Behavior, University of Kentucky, Lexington, KY, USA; 3 Gatton School of Business and Economics, University of Kentucky, Lexington, KY, USA; 4 College of Medicine, The Ohio State University, Columbus, OH, USA; 5 College of Public Health, The Ohio State University, Columbus, OH, USA

**Keywords:** Cancer screening, colorectal cancer, rural, health inequities, social networks, qualitative

## Abstract

Social networks influence health behavior and health status. Within social networks, “key players” often influence those around them, particularly in traditionally underserved areas like the Appalachian region in the USA. From a total sample of 787 Appalachian residents, we identified and interviewed 10 key players in complex networks, asking them what comprises a key player, their role in their network and community, and ideas to overcome and increase colorectal cancer (CRC) screening. Key players emphasized their communication skills, resourcefulness, and special occupational and educational status in the community. Barriers to CRC screening included negative perceptions of the colonoscopy screening procedure, discomfort with the medical system, and misinformed perspectives on screening. Ideas to improve screening focused on increasing awareness of women's susceptibility to CRC, providing information on different screening tests, improving access, and the key role of health-care providers and key players themselves. We provide recommendations to leverage these vital community resources.

We aimed to understand from the perspective of key players, or those individuals in a community optimally positioned to connect to numerous people (Borgatti, 2006), how to improve rates of colorectal cancer (CRC) screening among a rural population experiencing extensive health burdens. Given key players’ close connections to numerous people, such perspectives provide insight into how to decrease CRC screening barriers within a culturally acceptable, community context. Such insights are critical; despite screening guidelines to prevent or diagnose at an early stage, CRC remains the second leading cause of cancer mortality among males and females in the USA (American Cancer Society, 2014). In the Appalachian context, CRC mortality rates are higher partly due to lower CRC screening rates compared to average-risk adults in other geographic regions in the USA (Paskett et al., 2011) The CRC screening rate within US Preventive Services Task Force guidelines is estimated to be 53.1% in the USA, compared with 49 and 36.3% in Appalachia Ohio and Kentucky, respectively (Paskett et al., 2011; Reiter et al., 2013; Sallis, Owen, & Fisher, 2008). Complex and multifaceted reasons account for these disparities, perhaps best captured in the Social Determinants of Health Framework (Hemingway & Marmot, 1999; Weinstein, LaNoue, Hurley, Sifri, & Myers, 2015). These determinants include lower socioeconomic status, psychosocial factors (fear, knowledge deficits), and impeded access to medical care (Billings, 2006). The continuing CRC disparities that exist among this population have been identified by community residents and researchers as a high-priority area for intervention (Ely et al. 2014; Schoenberg, Hatcher, & Dignan, 2008).

While experiencing the burden of poor health, geographic isolation, and the lack of health-related resources, many Appalachian residents maintain strong social ties, social cohesion, and family and community loyalty (Coyne, Demian-Popescu, & Friend, 2006; Keefe, 1986). Leveraging such naturally occurring assets may comprise an acceptable and sustainable approach to encouraging preventive behaviors, including completing recommended CRC screening (Denham, 1996). Researchers increasingly acknowledge the pivotal role that social support and social networks play in shaping diverse health behaviors (Otero-Sabogal et al., 2010). Social networks often are defined as the structure of the social ties among a group of individuals that may or may not provide social support.

Social network analysis (SNA) involves understanding the connections and interactions among people through identifying individuals (actors/nodes) and the relationships among these individuals (ties) (Wasserman & Galaskiewicz, 1994 #7). SNA studies have demonstrated dense social networks and clustered behaviors among smokers (Christakis & Fowler, 2008), obese individuals (Mendoza, Drewnowski, & Christakis, 2007), and persons with HIV (Friedman et al., 2000 #9). Given clustering of individuals with similar health behaviors, it is likely that individuals who have completed cancer screening tests also cluster, and that individuals share networks, which are influential in directing behavior. Some evidence has supported, for example, the influence of a spouse on increasing CRC screening (Manne et al., 2012).

Within social networks, central actors or key players exert an influence over others’ behaviors and well-being (Wasserman & Galaskiewicz, 1994 #7). Key players are “those individuals receiving the most nominations in response to a network question such as ‘who do you go to for advice?’” (Valente, 2010). Indeed, a fundamental aspect of many behavioral social network interventions involves the identification of key players who can accelerate behavior change by delivering programs within their networks (Valente, 2010). Key players are connected to and embedded within a social network not only to enable dissemination of health information but also to help shape positive social norms about a health behavior (Borgatti, 2006).

The number of individuals that may be influenced by the key player depends on the structure and composition of the network in which the key player is embedded. Key players who are embedded in a network where all the individuals they communicate with tend to communicate with one other (dense network ties) appear to be better equipped to deliver a persuasive cancer screening message (Coleman, 1988). Being embedded in this tightly knit group, the key player may be more successful at changing the behavioral norms of the entire group. On the other hand, those key players, who are positioned among several groups of individuals in a network, who do not communicate with each other may have the ability to diffuse screening information to multiple groups (Valente & Pumpuang, 2007 #10; Valente, 2010 #4835). These types of key players may be better positioned to disseminate information to a broader array of individuals but may be less influential on the individual behavior of those in their network if they are not deeply embedded in their peers’ social worlds. In summary, key players embedded in tightly knit networks may be more influential on behavior, but those positioned among many diverse groups in a network may be better at disseminating information throughout the network (Manne et al., 2012).

We sought to understand how these individuals who were important in their social networks—that is, key players—might serve as to promote CRC screening. We contribute to the sparse research that characterizes key players in social networks by (1) identifying these key players, (2) gauging key players’ perspectives on the qualities or characteristics that make them key players, (3) obtaining their insight on CRC screening, and (4) capturing key players’ ideas for behavior change.

## Research design

This mixed method, sequential design involved egocentric social network data collection and analysis described below (Borgatti, 2006 #1). For the in-depth interviews with key players, we drew on qualitative standards of credibility (confidence in the “truth” of the findings accomplished through prolonged engagement in the research environment, peer debriefing, and member checking), transferability (indicating that the findings are applicable to other contexts, established by memoing, case study development, and, ultimately, thick description), dependability (demonstrating the capacity for the findings to be repeated and remain consistent accomplished through engaging in inquiry audits), and confirmability (whether our participants shape our findings rather than researcher bias or preconception determined by maintaining an audit trail and engaging in reflexity among research team and participants) (Lincoln & Guba, 1985).

## Human subject protection

All study procedures were approved by the institutional review boards of The University of Kentucky and The Ohio State University, protocol #11-0376-P3H, in accordance with the Helsinki Declaration. Prior to enrollment in the study, the rights and responsibilities associated with this project were explained to each eligible participant. Interviewers, all of whom had successfully completed the official Collaborative Institutional Training Initiative (CITI) course, responded to questions or concerns. Given low literacy rates, the official informed consent document was read to all participants. The main concern for this research involved insuring confidentiality of responses; we have pledged that all of the participants’ data would be entered into a password-protected database only accessible to researchers. The interviewer and participant then signed the document, and a copy was left with each party.

## Methods

### Overview

Three waves of data were collected. We asked the participants in the first wave (seeds) to name up to nine people (alters) whom they trust, who provides health information, and with whom they are in contact with on a regular basis, presumably those individuals most impactful in their CRC screening decision making. We repeated this protocol for the next wave of participants, allowing us to develop social network configurations of a sample of Appalachian residents and to identify those potential impactful individuals (key players) who were mentioned repeatedly by seeds and alters. For this paper, we focus on those individuals of importance to these social networks.

### Study setting

Two central Appalachian states were selected for this project to increase generalizability. Local Ohio and Kentucky staff recruited the first wave of participants (seeds) in two ways. First, flyers were hung in community locations (health department, library, etc.) and those interested could call project offices to be screened. Second, local community partners (members of our Community Advisory Board, etc.), who had extensive contact with diverse community members, were encouraged to invite potentially interested individuals to contact our project offices.

### Sample

Eligibility criteria for the seeds included: 1) 50+ years of age; 2) a resident of Appalachia Ohio or Kentucky; 3) able to speak and understand English; and 4) able to provide written or verbal consent to participate in the project. Recruiting individuals ages 50 and older allowed us to consider adherence to CRC screening for average-risk adults. Eligibility for the second wave (alters) included: 1) being 18+ years of age; 2) agreeing to participate in an interview; and 3) living in Kentucky or Ohio to enable in-person interviewing. The seeds had provided the following information on their alters: 1) age; 2) sex; 3) education level; 4) relationship to the seed; 5) residence; 6) how often they see/talk to the alter; 7) whether they give or get health advice from the alter; 8) whether they trust the health advice the alter gives; 9) whether they ever talked to the alter about colorectal, breast, cervical, and/or prostate cancer screening; 10) alter's cancer screening status (gender and age-specific screening for colorectal, breast, cervical, and/or prostate cancer); and 11) contact information. This information was verified with the alters, in-person interviews were scheduled, and informed consent was obtained. This entire process was repeated for a third round of up to nine alters.

### Key players

Consistent with established social network protocols (Borgatti et al., 2006 #1), we identified the key players’ identification through a two-step process. First, upon completion of the in-person interviews, two members of the research team conducted interviews with the interviewers to determine which individuals were mentioned frequently by numerous community members. Second, we used social network visualizations to illustrate and verify these individuals’ positions in the network. For this second step, participants provided verification of the first and last names of alters to identify any nicknames or misspellings that may have resulted in misplaced links between alters and would lead to inaccurately changing the structure of the network. We entered this verified list of all seeds and the two waves of alters into UCINET 6.504 software for SNA (Borgatti, Everett, & Freeman, 2002). We then created social network maps in NetDraw 2.134 software for social network visualization (Borgatti, 2002) and developed a network map from the connections among respondent's from each state. We selected this software as it is particularly useful in facilitating egocentric or personal social network development data, can handle large data sets with ease, and offers graphics that aid in visualization. Additionally, one of the investigators (AJ) worked in close collaboration with software developer Borgatti, allowing him to optimize the use of the program. After we developed the network visualizations, we verified with our interviewers the names of individuals who were mentioned to be particularly influential to determine their status as key players.

### Procedures

First wave (seeds): Trained interviewers conducted in-depth interviews at local community public locations (libraries) or homes. Following informed consent procedures, interviews focused on CRC screening behaviors and determinants. Additionally, these first participants (seeds) provided up to nine names of people (alters) with whom they spoke with most often or spent the most time with in their life. Finally, interviewers asked seeds which of these second level people (alters) knew each other among the nine individuals. Second wave (alters): For this second wave of data collection, we contacted the alters by telephone, told them the purpose of the study, and asked if they would be interested in participating in the study. Alters who agreed to participate then were screened for eligibility.

### Key players

The interviewers re-contacted each identified key player and asked to participate in a final in-depth interview to obtain his or her perspectives on characteristics of key players, perceptions of CRC screening, and ideas for future interventions to promote CRC screening. The interview guide is listed in Table I. Each key player interview was conducted in a participant's home or at a community site, lasted 45–60 min, and was audio recorded. None of the key players declined to participate. We provided a modest monetary incentive consistent with a standard 1-h interview honorarium in this region. The study was conducted from July 1, 2011 to May 2013, and the data were analyzed from April 2013 to September 2014.

**Table I:** Semi-structured interview guide for key players.

Please tell me a little about yourself. Probes: Where are you from? How long have you lived here? What's your family like? Many people have mentioned your name in the community as a person they talk with quite a bit. Can you tell us what qualities you have that make you such an important person in people's lives? Many people also mentioned that you are the person from whom they would get information about cancer screening. What do you think makes you a good person to get information from? As you probably know, in our community, we have a high rate of colorectal cancer. One of the ways to prevent cancer is by having regular screenings. And yet, most people here do not get screened. Probes: Why do you think this is? And why do you think those who do get screened are able to do so? Research has shown that people like you—those who interact with many others and are seen as a good information source—might help to get more people screened. As a person who has a lot of influence on others, I want to ask you a couple of questions. For those people you know who did get CRC screening, what encouraged them to get screened? Was there a particular person or program that encouraged them to get screening? What ideas do you have for increasing cancer screening? That is, what programs or interventions might be the most useful to encourage people to get screened? What sort of programs or interventions do you think would not work? Why not? What other ideas do you have to increase CRC screening here in our community? If costs were no consideration, are there programs or ideas you have to increase CRC screening?

### Analysis

We professionally transcribed each key player's interview verbatim and conducted content analysis, searching within the transcripts for recurring words and themes (Hsieh & Shannon, 2005) Broadly, our analytic goal involved identification of “core consistencies and meaning” (Patton, 2002, p. 453) by reducing and targeting the large volume of text to detect recurring words and themes. Two members of the research team read through all transcripts to identify primary themes to structure the codebook. During the second reading, both readers independently generated a list of codes that was then cross-checked with each other to produce coherent categories; the final codebook was approved by a third research team member. Then, a research team member engaged in line by line analysis of the transcripts, attaching codes. We undertook this data analysis without the use of a computerized program, hand coding all of the transcripts. During the process of coding, memos were developed in order to identify the relative frequency of the codes and the different contexts in which they emerged (Hsieh & Shannon, 2005). Themes that appeared across multiple participant transcripts are presented in the finding section, with attention to commonalities across participants’ responses. Where they appeared, discrepancies between participants were highlighted to indicate varying perspectives among the participants. An additional research team member reviewed the findings to confirm the accuracy of the final analysis.

## Findings

We identified several key findings. First, the 10 key players noted that qualities like strong communication ability, their own knowledge base, and a special status allowed them to serve as a resource for their network. These key players noted that negative perceptions of CRC screening, lack of familiarity with or access to the medical system, and inadequate or misinformed perspectives on screening undermined screening. They offered thoughts on CRC screening facilitators, including improved information dissemination, enhanced access to medical services, and a greater advocacy on the part of health-care providers. Additionally, the key players suggested that individuals who experience a significant health problem may be more inclined to obtain a preventive service like CRC screening and suggested that they—the key player—may play a role in promoting cancer screening. We first describe the networks and characteristics of the key players, then discuss each of these qualitative findings below.

### Key players’ networks

**Figure 1:**
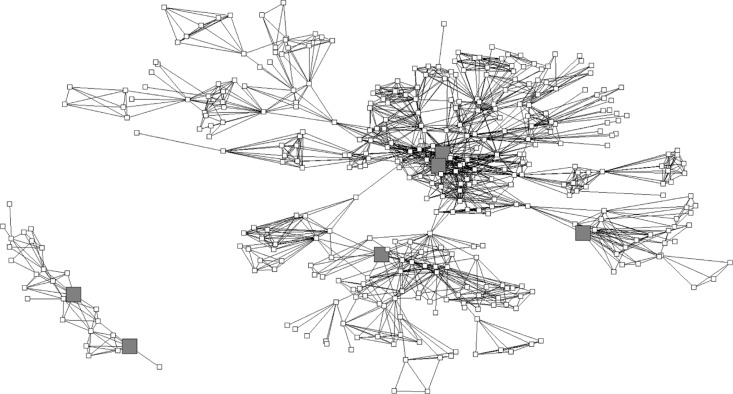
Visualization of Kentucky respondents’ social networks, *n=*395. Six key players. Squares represent Kentucky respondents; large gray squares represent key players.

**Figure 2:**
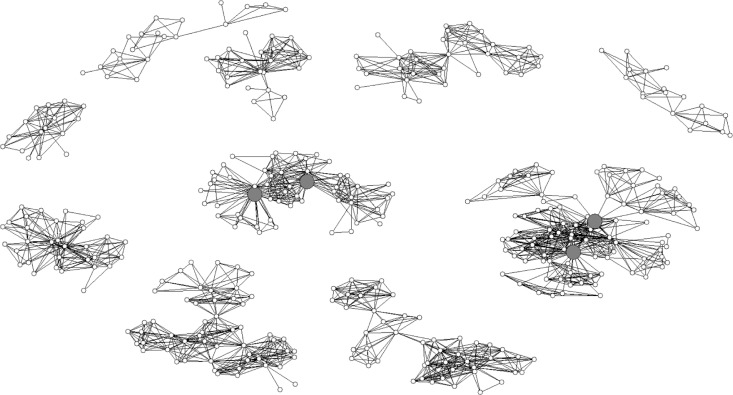
Visualization of Ohio respondents’ social networks, *n=*392. Four key players. Circles represent Ohio respondents; large gray circles represent key players.

Key players were connected to others and were identified as significant in their community's network. These networks, including all respondents, are visualized for Kentucky and Ohio in Figures 1 and 2, respectively.

**Figure 3:**
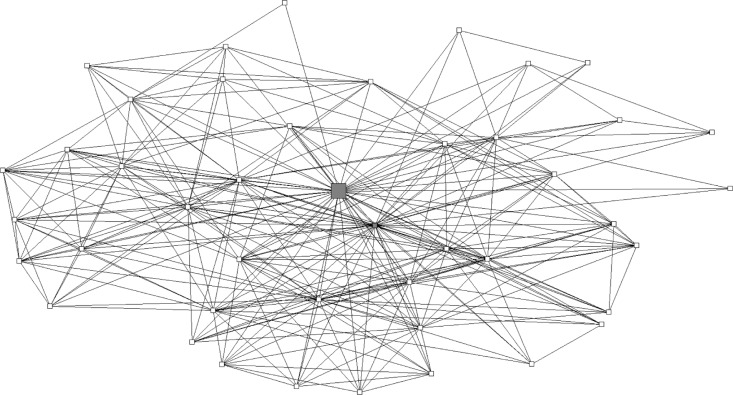
Example of a highly connected key player's personal network: key player A, *n=*44. Large gray square is key player A. Other squares are respondents in key player A's network. The small gray square is another key player who is in key player A's network.

**Figure 4:**
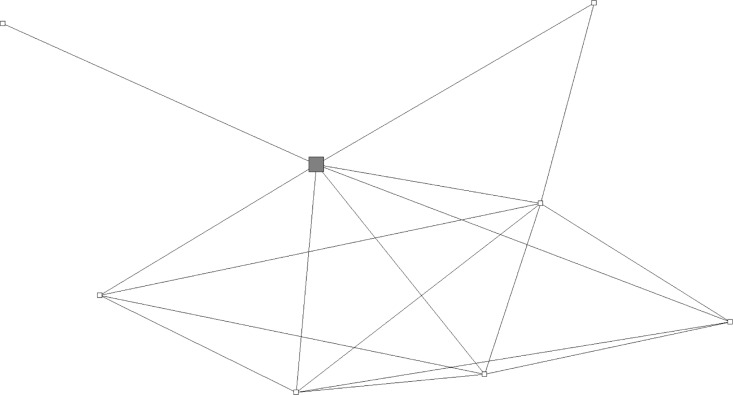
Example of sparsely connected key player's personal network: key player B, *n=*8. Large gray square is key player B, other squares are respondents who were linked with a tie. Many members in this network know one another, allowing for influence in the network, but the key player is not linked to many others in the larger community network.

Some key players had numerous connections to others, such as Key Player A shown in Figure 3, who was connected to 43 others. However, some, such as Key Player B (Figure 4), had sparser connections yet were still deemed influential by others in the network.

### Key players’ characteristics

Table II highlights the personal characteristics of the 10 key players identified from the four social networks.

**Table II:** Sociodemographic characteristics of key players (*n*=10).

Variable	Key players	% of key players
Gender		
Male	2	20
Female	8	80
State		
Ohio	4	40
Kentucky	6	60
Race		
White	10	100
Household income		
<$20,000	1	1
$20,001–$30,000	1	10
$30,001–$40,000	0	0
$40,001–$50,000	4	40
$50,001–$60,000	0	0
$60,001–$70,000	2	20
$70,001–$80,000	2	20
Education level		
High school diploma	5	50
Some college	3	30
College degree	1	10
Graduate or professional degree	1	10
Married/living with partner		
Yes	9	90
No	1	10
Completed colorectal screening		
Yes	3	30
No	7	70
	Key player, mean	Key player, SD
Age	54.2	6.2
Years lived in county	43.8	21.1
Self-rated health (1=poor, 5=excellent)	2.4	1.0
Social network ties to others in the community	24.1	12.8

### Key players’ roles and features

Key players suggested they play this role because of their communication skills, influence, and personal experiences. Participants described the interactions that positioned them in influential positions within health information networks, although many key players were modest or self-effacing in identifying what they believe made others consider them an important person in their social networks.

Key players remarked that their communication style, essential character, and background explained their key role in social networks. Many key players described that their communication style was appealing to others, particularly those seeking health information. Several people highlighted their caring, friendly nature that made them important to others. An ability to listen was appealing to those who sought them out: “people like to be listened to without being judged and without being told what to do.” This way, they could “make up [their] own minds.” One woman explained that her resourcefulness was an asset. The people with whom she worked told her, “if they have a question, they know that they can come to me … [and] if I don't know the answer, I will find them the answer.” The way that key players presented information and resources also made a difference. According to one key player, “I think they may value my opinion because … I'm really a blunt person; I don't sugarcoat a lot of things. It's like the old saying: ‘if you don't want to know, don't ask’.” Other key players advocated for sharing information without pressure; one key player commented that her nursing practice put an emphasis on “educating the patient so that they can make an informed decision.”

Many others speculated that they occupy a key player role because their position in the community—as a firefighter or as a pastor's wife—made them known to many people. In other cases, being a health-care practitioner, being related to one, or having experienced a health crisis or engaged in screening made them knowledgeable about health to others. Explaining her status as a key player, one nurse commented, “For the past 12 years I've worked at the health department and so I've worked with a lot of people over the years, met a lot of people, and, and I guess that's why.” Having a family member or close friend who had cancer, one key player explained, made others feel that she might relate to their own questions about cancer and cancer risk: “I guess unless you've gone through it or close enough to somebody that's gone through it, you really don't realize how it can impact them.”

### CRC screening barriers, according to key players

Key players emphasized that the primary barriers to completing CRC screening among the people they knew involved: a) negative perceptions of the colonoscopy procedure, b) avoidance of thinking about CRC and of engaging with the health-care system more generally, c) having information—but avoiding screening anyway, and d) structural constraints.

### Barriers: negative perceptions of the colonoscopy procedure

Nearly all the key players stated that people they knew found CRC screening to be an embarrassing, problematic procedure. They drew attention to aspects of the procedure that they found offensive and invasive. As one woman put it uncomfortably, “who likes the idea of somebody sticking something up their bottom? That's not a good idea.” Because the specifics of the procedure were vague or uncertain to many people, some expressed concern that getting a colonoscopy could adversely affect their other health conditions. As one woman put it, “I had that hernia and I know that colonoscopy goes up through there and I don't know what my hernia entails. So I've had I've just had nightmares of them going up there with that scope and busting something ….” The time and discomfort entailed in the preparation for the procedure was also described as a barrier that turned many off from pursuing screening.

In the rural areas where these key players live, the potential embarrassment caused by the procedure was exacerbated by the likelihood that they might know the medical staff conducting the procedure. One key player who was a nurse elaborated,

I've had people that I work with say that they would rather go someplace else if they were going to have to have surgery or any type of procedure because they know the OR [operating room] staff…and they have to see those doctors every day face to face.

Many key players had heard frightening stories about others’ experiences that were a deterrent. One woman explained,

They wanted my mother to have a colonoscopy but they didn't want to put her to sleep to have it. So there's some scary stories out about that; not being put to sleep to have a colonoscopy is not a pleasant thing.

In contrast to this woman's perspective, others commented on their fear of anesthesia during the screening—or, that the anesthesia would wear off mid-procedure.

### Barriers: people don't want to think about CRC

Every key player explained that the people they knew did not want to consider their own risk for CRC. Though they might be aware of CRC, one woman said, “I think a lot of people, it's like you know if I don't claim it's not going to happen.”

Key players frequently observed that many of the people they knew felt that screening was unnecessary unless they exhibited harmful symptoms. One person characterized this thinking as: “If I don't hurt and I'm not in pain and there's nothing wrong and I go to the bathroom when I'm supposed to and I do this when I'm supposed to so I'm okay.” CRC screening became an interest only once their health changed significantly:

When people have stomach problems or they're having bleeding from their bowels or something like that they're real interested in getting screened but I think that the mindset of most people is if they're not having any problems they don't need to be screened for colorectal cancer.

Most key informants related screening avoidance to a general distrust of doctors and a preference for taking care of themselves on their own. “I think a lot of it is cultural; you know it's: you don't go to the doctor, you treat it at home.”

Key players also expressed the concern that CRC screening was misperceived as more important for men than women:

I thought of it as more of a male problem than a female problem … I don't know if they stress it like in the advertisements or whatever, they stress it more for the males to get the colonoscopy and they stress for the females to get the Pap test for the cervical cancer.

### Having information—but avoiding screening anyway

While most key players argued that a lack of information was a barrier to screening, many admitted that they did not obtain CRC screening even when they were well informed, including having received a recommendation from someone in their social network. Several key players who were health-care providers admitted that, even though they were aware of the need for screening, they themselves had not gone through with it. As one woman explained,

I'll be honest because of my age, I've been recommended to have a colonoscopy and I said no. And I'm in the health care profession; I see how easy if it was caught early you know that colorectal cancer can, you know it's curable if it's caught early enough.

### Barrier: logistical and structural constraints

Key players explained that various structural factors potentially impeded receipt of CRC screening. These constraints included the cost of cancer screening and a lack of insurance coverage. For some, a lack of transportation was a barrier. One woman noted, “You have to go away from here to do it. That's one thing that keeps people from doing it if they don't have family that can take them.”

### CRC screening facilitators according to key players

Key players maintained that CRC screening was facilitated by changes in the availability of health care and the salience of significant health problems. Both being able to afford the cost of the procedure and having insurance coverage were crucial to enabling the receipt of colonoscopies. To one key player, such affordability made all of the difference in the rates of screening: “I think with the way that the insurances have been … where they have been like they pay for all of that well preventive care and all of that cancer stuff but I think that has really helped a lot.” Others explained that access to screening was more available in this rural region than previously and had become incorporated into doctors’ care. One woman explained,

the doctors we used to have, they felt like they were the be all and end all and they didn't want to refer you to other doctors, to specialists; they thought that what they were doing was good enough and that they'd throw some pill at you to take and didn't care whether you got screenings or not.

Most key players acknowledged that having a doctor's referral facilitated the colonoscopy: “That's it; you just do it because the doctor said to.” At the time of the interviews, the Affordable Care Act (ACA) had just been rolled out in the state and, with it, opportunities for preventive services like colonoscopy increased dramatically. However, many of the key players were aware of the impact of the ACA, including how the program expanded insurance coverage in the region, and covered CRC screening (www.kff.org/interactive/implementation-timeline/). As a result, most participants did not directly comment on the ACA.

### Facilitator: relevance of screening because of a significant health problem

Whether their own or someone important to them, key players indicated that learning about a health problem motivated them to get screened. Indeed, half of the key players said they knew people whose screening was initiated by the appearance of serious health problems. For others, seeing people they knew suffer from cancer motivated them to complete screening. One woman described how health problems that emerged in several people in one social network influenced the others in the group to seek out screening. Before,

nobody really … took care of their health or went to the doctor as much. After three of them became sick, from there it was like “oh wow, we need to take care of this,” and … people started taking a little bit better care of their health.

### Approaches that may improve CRC screening, according to key players

Key players had two primary suggestions to increase CRC screening: a) develop and increase the information available about the need for screening and specifics about the screening procedure and b) enhance the means through which screening can be facilitated by the health-care system.

### Promising approach: increase CRC screening information

To address the challenge that screening provokes fear and often is avoided by the people they knew, key players concentrated on ways to build awareness about CRC risk and the specifics about the procedure:

Like with cigarettes, they've got pictures of people's lungs that have smoked for however many years … Like, this is what your lungs look like, and if you quit smoking this is what your lungs will look like. You know within so many days, your lungs are starting to clear up and they're not going to be so cloudy as before. But do they even have something like that for colon cancer? Do they have pictures of like this is what it looks like before and this is what it looks like after you get cancer?

Basic education about the procedure could reduce widespread reticence. One person suggested, “have an old probe and show them, hey it's not that bad.” The majority of key players agreed that people who have undergone colonoscopies themselves could be the most effective for promoting the importance of completing the procedure. For some people “talking to other people that have had one done,” one woman explained, those who are hesitant to be screened would “see that it's not that bad and after it's over, you really don't even know you've had it done.” Others felt that the experience was too negative and embarrassing for them to advocate for CRC screening.

According to several participants, the key to programmatic success involves integrating health information into established programs within local institutions. Multiple people mentioned the surge in awareness about other types of cancer from booths, health fairs, and outreach—but that CRC was almost always left out of these campaigns. The success of previous cancer education events, they reasoned, meant that health fairs could offer a positive location for educational enhancement. Key players suggested that locating educational events in comfortable and convenient locations—like senior centers, area restaurants, churches, and libraries—could facilitate greater comfort with a topic about which many people feel uncomfortable or embarrassed. But even the benefits provided by these locations had their limits. As one woman explained, “Well when you're talking about the senior center or a church group, that's like a comfort zone. And when it's open to the community and it's not really in your comfort zone, I'm … I don't know like how open people would be.” To combat widespread aversion to discussion about CRC and screening, she advised that health educators could integrate CRC screening messages into other health prevention programming.

Some female participants suggested that, from such sources of information, awareness about the need for CRC screening would be spread by word of mouth. This would be particularly useful for women, one woman explained, because, “Women do tend to ask other women.” But several offered caution about the limits of depending on word of mouth for men:

… when us women get together we talk about everything but you just never hear about the men and I just don't know what you can do to get them, to approach them and get them to talk I just don't see … I just don't see them sitting down and talking about something like that.

Though many key players had mentioned that a fear of doctors was a barrier to screening, many argued for an active role of doctors in facilitating screening. One woman suggested that, “I think he should be the one who's pushing it a little more I think for people to get screened. I mean he can't make you do it but he could strongly suggest that you have this done.” Another woman offered that the elevated social position of doctors would make certain groups receptive to their advice: “I would say particularly with that age group, like seniors, that you would need to have a professional person because there is especially in that generation what do I want to say … a respect for people.”

### Promising approach: expand health-care opportunities

Key players recommended how expanding health-care availability could facilitate screening. Such an expansion might include increasing the availability of specialists in the region who could perform the screening and deploying mobile clinics to reduce transportation barriers. Increasing the affordability of colonoscopies—and ideally, incentivizing it—was felt to address the economic barriers to screening. Several people mentioned the home stool test test as a screening method that was less invasive and that people might be more willing to do.

### What would not work, according to key players

Several key players described what would *not* facilitate screening. In spite of the need for screening, they explained, it needs to be the choice of individuals: “Just don't try to force anybody to do it. Just let everybody make their own decision. It's their body.”

## Discussion & implications

### Key players’ characteristics

This study contributes novel perspectives on the role of key players for health promotion in social networks. The social network maps produced from this study suggest there are at least two types of key players within social networks. One key player existed within a highly interconnected set of alters or community members (Figure 3). On the other hand, a key player may be at the core of a more diffuse social network, potentially allowing the key player to reach a wider array of unconnected individuals as illustrated in Figure 4. Previous research has demonstrated that both types of key players may be successful change agents to promote cancer screening among members of their social network (Dearing, 2008 #11), (Valente & Pumpuang 2007 #10). Future studies should investigate using both types of key players as potential change agents in order to influence CRC screening behaviors, with the goal of implementing successful community interventions (Cutrona et al., 2015).

Key players highlighted several characteristics that they felt were fundamental aspects of connecting to people, including their communication ability and style, their status in the community, their resourcefulness, and knowledge base. The key players reported having a communication style that emphasizes the truth in a caring, nonjudgmental, and friendly manner. Additionally, consistent with other social network research, key players recognized that they were aware of local resources and individuals able to connect community members to cancer screening resources (Israel, Eng, Schulz, & Parker, 2005). Most (80%) key players were women, confirming broader literature demonstrating the role of women as central providers of health information (Warner & Procaccino, 2004). While participants indicated characteristics typical to women in the literature on social networks—communication skills and a central role in the community—it is interesting that none of our participants described the role of gender in facilitating their influence.

### Key players’ perspectives and recommendations to increase CRC screening

Our study findings suggest that numerous factors influence the uptake of CRC screening among a traditionally underserved population. Consistent with the Social Determinants of Health Framework (Hemingway & Marmot, 1999; Weinstein et al., 2015), our study has identified determinants that both impede and facilitate CRC screening, including the socioeconomic and political context, social position, and psychosocial circumstances.

The larger socioeconomic and political context, including the availability of health-care resources and the health policy environment, exerts a strong impact on screening. The absence of sufficient health-care access makes it less likely that patients will receive a recommendation to obtain screening. Other researchers have confirmed this result; using the nationally representative Health Information National Trends Survey found that respondents who discussed screening with a provider had 8.83 higher odds of CRC screening than those who did not discuss screening (95% CI, 7.20–10.84). For those who were recommended a screening modality by their physician after screening, the odds of CRC screening were 2.04 times higher than those who did not have a screening modality recommended (95% CI, 1.54–2.68) (Laiyemo et al., 2014). Within this larger category of social determinants, key players identified that improved health-care availability may remove a significant barrier for many individuals who previously had no insurance or who are underinsured. Although the ACA may assist in reducing CRC disparities, we have to be cautious because coverage for CRC may vary, depending on the state an individual resides in and whether the policy covers all screening tests and follow-up procedures (Green, Coronado, Devoe, & Allison, 2014).

Our findings also emphasize the key determinants of social position, including gender and socioeconomic status. Key players also mentioned CRC screening barriers that were especially relevant to the women living in their communities. The misperception that CRC mainly affects men has been mentioned frequently and has been previously reported among other medically underserved populations (Meissner, Breen, Klabunde, & Vernon, 2006; Rosenwasser et al., 2013).

Finally, psychosocial factors, including social cohesion, influence CRC screening. For example, key players also mentioned another social network-related screening facilitator, the motivation to complete a screening test because someone they knew completed CRC screening. The positive associations of social connections, social support, and CRC screening have been reported among varying populations. In the Framingham Heart Study, there was a slight increase in CRC screening if spouses were screened (Rawl, Menon, Burness, & Breslau, 2012). The 2005 Health Information National Trends Survey found that those who were socially isolated had a lower likelihood of getting screened for CRC (Ye, Williams, & Zu, 2009). Similarly, among a sample of African-Americans, those with more social connectedness, particularly from church, were more likely to report CRC screening (Kang & Bloom, 1993). In Japanese Americans, greater emotional support and network norms supporting CRC screening were associated with CRC screening rates (Honda & Kagawa-Singer, 2006). Consistent with these findings, key players emphasized that personal interactions with trusted community members may increase awareness of this equal opportunity cancer risk. Structured visits by key players serving as community health workers have demonstrated success in such interactions and improved cancer screening rates (Holt et al., 2009; Klabunde et al., 2011). In the Appalachian context, such community health workers represent a strong and readily available asset (Schoenberg, Howell, & Fields, 2012).

Frequently, several of these determinants comingle and reinforce each other. For example, cultural norms, like avoiding seeking medical care unless it is an emergency, derive from a long history in the rural regions of limited economic resources on a personal level and lacking access to medical care on a policy or macro level, leading to the cultural expectation of self-reliance (Doescher et al. 2009).

Conversely, as described in the Social Determinants of Health Framework and highlighted in our findings, factors such as social position, cultural norms, and social context may exert a positive impact on screening (Kawachi & Kennedy, 1997). For example, social cohesion among rural residents may elevate the role of influential others like key players. These individuals, who tend to know many people and who have garnered the trust of diverse community members, may be able to share information to increase the salience of screening. According to the key players in this study, rural residents may be more accepting of CRC screening when they understand the salience of CRC and learn strategies to overcome health-care professional shortages (Billings, 2006).

## Limitations

First, out of concern for confidentiality, some individuals refused to provide contact information for their alters; thus, we were not able to complete an interview of all alters named by each seed. This limitation may have directed us to incorrectly identify an individual as a key player within the network. However, the individuals identified through the network analysis tended to be well connected and they were mentioned as important individuals by several members within their network. Additionally, although we employed specific protocols to achieve the qualitative standards of achieving trustworthiness, the lack of an objective metric by which to measure our success leaves some room for differing interpretation of findings. Trustworthiness generally is considered to be comprised of four evaluative criteria (credibility, transferability, dependability, and confirmability). We attempted to achieve these standards through deliberate strategies, including audit trails, memoing, negative case analysis, and member checking (Morse, Swanson, & Kuzel, 2001).

Finally, we concede that the key players selected in Ohio and Kentucky Appalachia are not necessarily representative of all adults, even those residing in the Appalachian region of the USA. However, many of the characteristics of our participants are similar to the regional demographics (low socioeconomic status, educational level, etc.). Finally, we acknowledge that these networks were started from a convenience sample of seeds from both states.
